# Renewable energy transition and the local economic development of lagging-behind regions: Evidence from the offshore wind energy industry in the UK

**DOI:** 10.23889/ijpds.v8i2.2304

**Published:** 2023-09-14

**Authors:** Enrico Vanino, Thomas Siddall, William Rupp

**Affiliations:** 1 University of Sheffield, Sheffield, United Kingdom

## Objectives

In this study we analyse the potential impact of the renewable energy transition on the local economic development of lagging-behind regions, focusing in particular on the causal impact of offshore wind farms on the economic development of local onshore coastal communities in the UK, which are usually deprived left-behind regions.

## Methods

We consider the current impact of this transition on the economic development of places, analysing employment and productivity growth in local industries directly affected, as well as the magnitude of potential externalities along the supply-chain. Linking publicly available data on offshore wind farms, offshore wind suitability, and local economic outcomes, we develop an instrumental variable approach to address endogeneity, modelling the probability of wind farms’ locations based on offshore natural characteristics, such as water depth, distance to shorelines, and wind speed factors.

## Results

This is the first study providing empirically robust analysis at a granular level on this topic. After mapping the location of offshore wind farms and link these to onshore local communities through the location of ports and other infrastructures servicing these farms, we estimate a significant positive effect on local employment and productivity growth in the offshore wind energy sector, as well as the indirect effect along the supply chain.

## Conclusion

The rapidly growing renewable energy sector could become a key player in the local economic development of peripheral and lagging-behind regions. This could allow many peripheral regions across developed countries, once dependent on mature declining industries, to regenerate their local economies by sustainably transitioning towards new higher added-value green industries.

